# *Lankesterella* (Apicomplexa, Lankesterellidae) Blood Parasites of Passeriform Birds: Prevalence, Molecular and Morphological Characterization, with Notes on Sporozoite Persistence In Vivo and Development In Vitro

**DOI:** 10.3390/ani11051451

**Published:** 2021-05-18

**Authors:** Carolina Romeiro Fernandes Chagas, Josef Harl, Vytautas Preikša, Dovilė Bukauskaitė, Mikas Ilgūnas, Herbert Weissenböck, Gediminas Valkiūnas

**Affiliations:** 1 Nature Research Centre, Akademijos 2, 08412 Vilnius, Lithuania; vytautas.preiksa@gmail.com (V.P.); dovilebu7@gmail.com (D.B.); ilgunasmikas@gmail.com (M.I.); gediminas.valkiunas@gamtc.lt (G.V.); 2 Institute of Pathology, University of Veterinary Medicine, Veterinärplatz 1, 1210 Vienna, Austria; josef.harl@vetmeduni.ac.at (J.H.); herbert.weissenboeck@vetmeduni.ac.at (H.W.)

**Keywords:** *18S* ribosomal RNA, *Lankesterella*, birds, development in vivo and in vitro, molecular and morphological characterization, phylogeny, *Hepatozoon*

## Abstract

**Simple Summary:**

Birds are hosts of various apicomplexan blood parasites, whose blood stages are often similar, resulting in much ongoing debate about the taxonomic and genetic identity of some species. Parasites of several closely related apicomplexan genera can be distinguished mainly by differences in their life cycles in both vertebrate and invertebrate hosts. Recent studies confirmed that some avian blood parasites, which were formerly attributed to the genus *Hepatozoon*, are genetically closely related to the amphibian parasite *Lankesterella minima* and might belong to the genus *Lankesterella*. To understand the distribution and diversity of avian *Lankesterella* parasites, we examined samples from wild birds, combining molecular genetics and microscopic methods. Experiments which aim for a better understanding of the life cycle of these parasites, and their host specificity, were designed. We demonstrated that avian *Lankesterella* parasites are more diverse than previously thought, and several species of *Hepatozoon* described in birds in fact belong to *Lankesterella*. Two new *Lankesterella* species parasitizing birds are described, and one species is re-described. This study contributes to a better understanding of diversity and distribution of bird *Lankesterella* spp. and shows directions for future research on their pathogenicity.

**Abstract:**

Recent studies confirmed that some *Hepatozoon*-like blood parasites (Apicomplexa) of birds are closely related to the amphibian parasite *Lankesterella minima*. Little is known about the biology of these pathogens in birds, including their distribution, life cycles, specificity, vectors, and molecular characterization. Using blood samples of 641 birds from 16 species, we (i) determined the prevalence and molecular diversity of *Lankesterella* parasites in naturally infected birds; (ii) investigated the development of *Lankesterella kabeeni* in laboratory-reared mosquitoes, *Culex pipiens* forma *molestus* and *Aedes aegypti*; and (iii) tested experimentally the susceptibility of domestic canaries, *Serinus canaria*, to this parasite. This study combined molecular and morphological diagnostic methods and determined 11% prevalence of *Lankesterella* parasites in Acrocephalidae birds; 16 *Lankesterella* lineages with a certain degree of host specificity and two new species (*Lankesterella vacuolata* n. sp. and *Lankesterella macrovacuolata* n. sp.) were found and characterized. *Lankesterella kabeeni* (formerly *Hepatozoon kabeeni*) was re-described. *Serinus canaria* were resistant after various experimental exposures. *Lankesterella* sporozoites rapidly escaped from host cells in vitro. Sporozoites persisted for a long time in infected mosquitoes (up to 42 days post exposure). Our study demonstrated a high diversity of *Lankesterella* parasites in birds, and showed that several avian *Hepatozoon*-like parasites, in fact, belong to *Lankesterella* genus.

## 1. Introduction

Blood parasites are extremely common in birds and other vertebrates [1]. Some of them occur in blood plasma, such as *Trypanosoma* spp. and microfilariae of filariid nematodes, while others are intracellular (develop in blood cells) [2]. The intracellular parasites are highly diverse and belong to several taxa of the phylum Apicomplexa, such as the orders Haemosporida (e.g., common genera are *Plasmodium*, *Haemoproteus* and *Leucocytozoon*), Piroplasmida (e.g., *Babesia*, *Theileria* and *Sauroplasma*), and Eucoccidiorida, the latter featuring the two suborders Adeleorina (e.g., *Hepatozoon*, *Haemogregarina* and *Karyolysus*) and Eimeriorina (e.g., *Lankesterella*, *Schellackia* and *Lainsonia*) [1,2,3]. In the past, these parasites were described mainly using morphological features, but also based on their occurrence in certain host species. However, the blood stages of closely related parasites belonging to several genera are often similar, which resulted in some confusion and still ongoing taxonomic debates about the validity of many species names and their generic position. This is particularly true for *Hepatozoon*, *Isospora*, and *Lankesterella* parasites in birds [1,3,4,5].

Species of *Hepatozoon*, *Isospora*, and *Lankesterella* belong to the order Eucoccidiorida (subclass Coccidia, class Conoidasida, Apicomplexa), and even though they can be readily distinguished due to differences in their life cycles, their blood stages are often remarkably similar and present in the same types of host cells. *Hepatozoon* parasites are obligate heteroxenous parasites, with various invertebrate vectors (ticks, mites, tsetse flies, reduviid bugs, sand flies, and mosquitoes) acting as definitive hosts. A syzygy formation occurs in vectors before the sexual process, and oocysts finally develop. The latter contains numerous sporocysts with sporozoites. In the vertebrate host, the merogony takes place in different tissues, and gamonts finally appear in the circulating erythrocytes and/or leukocytes. Transmission of *Hepatozoon* parasites occurs when the infected vector is ingested by a susceptible vertebrate host; this is also sometimes possible by predation of one infected vertebrate by another vertebrate host (for example, when a snake eats an infected rat) [1,3].

The life cycle of *Lankesterella* parasites is different [3]; it occurs entirely in the vertebrate hosts, in which merogony, gametogony, and sporogony take place and invasive sporozoites develop. The latter persist in blood cells. Various blood-sucking invertebrates (leeches, mites, and mosquitoes) can serve as the paratenic vectors, in which parasite sporozoites persist. As in *Hepatozoon* infection, the transmission between vertebrate hosts occurs orally by ingestion of infected vectors [3,6]. In the past, several authors attempted to investigate the life cycle of *Lankesterella* species in birds, however, based on the limited experimental observations and illustrations of blood stages, some studies were likely dealing with *Isospora* (synonym *Atoxoplasma*) parasites, but not *Lankesterella* infections [7,8,9,10]. Due to this uncertainty, further investigation of the life cycle remains important in order to better understand the biology of *Lankesterella* spp. and to distinguish them from *Hepatozoon* parasites [11].

Several species of *Hepatozoon* were described and subsequently often reported in many bird species. However, most of these reports were based exclusively on morphological and morphometric parasite features, and vertebrate host identity [12,13,14,15,16,17,18]. Based on molecular diagnostics, *Hepatozoon* infections are certainly present in birds [19]. However, recent molecular studies also indicate that some avian parasites, which have been formerly believed to be *Hepatozoon* spp., are closely related to the amphibian parasite, *Lankesterella minima*, but not *Hepatozoon* species [4,5,20]. This calls for further molecular and morphological characterization and comparison of these neglected avian infections.

To add new knowledge about avian *Lankesterella* parasite biology, this study aimed (i) to determine the prevalence of infection in adult and juvenile Passeriformes birds of several species, using molecular-based methods; (ii) to perform morphological and molecular characterization of *Lankesterella* parasites in naturally infected birds; (iii) to investigate the development of *Lankesterella kabeeni* in laboratory-reared mosquitoes *Culex pipiens* forma *molestus* and *Aedes aegypti*; and (iv) to experimentally test the susceptibility of domestic canaries, *Serinus canaria*, to *L. kabeeni* infection.

## 2. Materials and Methods

### 2.1. Blood Sampling

Passeriformes birds were caught with mist nets, zigzag traps, and a big Rybachy type trap at Ventės Ragas Ornithological Station, Lithuania (55°20′28.1″ N, 21°11′25.3″ E). Samples from adult birds were obtained during the spring after bird arrival from African wintering grounds (May 2015–2019). Samples from juvenile Acrocephalidae species were also collected and processed; this group of birds was chosen based on the high prevalence of parasites found in the adult individuals belonging to the same family. Samples from juveniles were collected during the summer (July 2016–2018). Bird species and age were identified by professional ornithologists at the bird ringing station.

Blood samples were taken from the brachial vein using a sterile needle and heparinized capillary tubes. Approximately 50 µL of blood was collected, and few drops were used to immediately prepare blood smears, which were fixed in absolute methanol and stained with a 10% Giemsa solution [21]. Remaining blood was stored in SET buffer (0.05 M tris, 0.15 M NaCl, 0.5 M EDTA, pH 8.0) for posterior molecular analysis; these samples were maintained at 4 °C in the field and stored at −20 °C at the laboratory.

### 2.2. Microscopic Examination

Infected birds were determined using PCR-based testing of blood samples, and blood films of the PCR-positive samples were examined under microscope. In order to perform morphological analysis of *Lankesterella* parasites, blood films containing different lineages were selected based on DNA sequence information; these preparations were carefully examined at 1000× magnification (oil immersion) using an Olympus BX41 light microscope equipped with an Olympus DP-12 digital camera and the Olympus DP-Soft v. 3.2 imaging software (Olympus, Tokyo, Japan). The intensity of parasitemia was determined by counting the number of parasites per 100 thrombocytes or leukocytes. Illustrations and measurements were prepared using the same instruments. Representative blood films were deposited in the Nature Research Centre, Vilnius, Lithuania and in the Queensland Museum, Brisbane, Australia (see parasite descriptions for accession numbers of these preparations).

### 2.3. In Vitro Development

In vitro development experiments were conducted during fieldwork at Ventės Ragas Ornithological Station in May 2018. An intensively naturally *Lankesterella* infected sedge warbler *Acrocephalus schoenobaenus* (AS1) (donor of parasite blood stages, sporozoites) was selected using the buffy coat method as described by Chagas et al. [22] (Table 1). The intensity of parasitemia was 53% or 53 sporozoites per 100 thrombocytes. Approximately 75 µL of blood was collected from the brachial vein and immediately mixed with a 3.7% solution of sodium citrate, in a proportion of 4 parts of blood to 1 part of sodium citrate (for a detailed description of this method see Valkiūnas et al. [23]). This mixture was exposed to air and maintained in a humid chamber at room temperature (~22 °C). At a set of time intervals (1, 3, 5, 10, 15, 30, and 45 min and 1, 2, 3, 4, 6, 8, 10, 12, and 24 h) after exposure of blood to air (EBA), the mixture was gently mixed, and smears were prepared on ready to use glass slides. These preparations were fixed, stained as blood films, and examined under the microscope at 1000× magnification (oil immersion) for 15–20 min using the same equipment as described above. Representative preparations of in vitro parasite stages were deposited in the Nature Research Centre, Vilnius, Lithuania (collection numbers 49271NS-49275NS).

### 2.4. Mosquito Experimental Infection and Dissection

Two naturally infected sedge warblers *Acrocephalus schoenobaenus* (AS1 and AS2, Table 1) were selected as donors of *Lankesterella* parasites for exposure of laboratory reared *Cx. pipiens* forma *molestus* and *Aedes aegypti*. These experiments were designed to follow the development of the parasite in mosquitoes and the effects on their potential vector. Two non-infected *S. canaria* (SC1 and SC2) were used as intact controls (Table 1).

The methodology used to expose mosquitoes was described by Kazlauskienė et al. [24]. Briefly, the bird was placed in a plastic tube containing a slit, which was used to fix the birds’ legs. Next, both tube ends were covered with silk mesh. The tube with the bird (donor or control) was placed into a cage containing ~30 mosquito females; this bird was kept in the cage for 10–20 min, and the mosquitoes willingly took blood meals. The fully engorged females were collected and transferred to a smaller cage (12 cm^3^) where they were maintained at room temperature for up to 42 days post exposure (DPE), or until all infected insects died, and used for dissections and/or molecular analysis. The insects were fed with a 10% sugar solution daily. Experiments with infected and non-infected birds were done separately using the same design.

Experimentally infected and control mosquitoes were dissected every 2–3 DPE. The number of dead females found inside each group of the experiment was registered daily. The observation period finished when all females were dead in each group (Table 1). To prevent contaminations, dissection needles were disinfected in fire after each dissection. Before dissection, insects were anesthetized using 96% ethanol vapor from a moistened cotton ball. The first step in the mosquito’s dissection protocols consisted of separating head and thorax from the abdomen and placing them separately in a small drop of 0.85% physiological saline. Next, the head and thorax were smashed and covered with a coverslip. The midgut was extracted from the abdomen and covered with a coverslip. All dissected body parts were examined microscopically for the presence of parasites. After examination of the entire midgut, it was also smashed by gently pressing the cover slip against the glass slide, and the midgut content was also examined. Each vector preparation was screened entirely at 200× and then at 400 magnifications using the same microscope equipment as described above. After examination, the coverslip was removed, and preparations were air dried out at room temperature, fixed with absolute methanol, and stained with 4% Giemsa solution for 1 h. Residual parts of all dissected and naturally dead mosquitoes were fixed in 96% ethanol and used for PCR-based analysis to confirm the presence of *Lankesterella* parasites. Representative preparations of mosquito midgut were deposited at the Nature Research Centre, Vilnius, Lithuania, for each experiment (collection numbers 49276NS-49284NS).

### 2.5. Serinus canaria Experimental Infection

To test susceptibility of *S. canaria* to *Lankesterella* parasites, the birds were experimentally exposed using three different approaches. Each *S. canaria* received one procedure in all experiments. First, the canaries were forced to swallow infected mosquito midguts. This was done by dissecting six *Ae. aegypti* mosquitoes that were exposed to infected *A. schoenobaenus* AS1 (12 and 20 DPE). The mosquito midguts were isolated and gently crushed in an Eppendorf tube with a few drops of 0.85% physiological saline. This mixture was separated into three approximately equal parts; two of them were used to feed two *S. canaria* directly on the beak (one part each), and the third part was used for molecular confirmation of the presence of *Lankesterella* parasites, as described below. The same procedure was performed with the control birds, however non-infected mosquitoes were used from the intact mosquito colony.

Second, canaries were fed with infected blood that was collected from *A. schoenobaenus* AS1 (Table 1). Briefly, approximately 40 µL of blood was collected from this bird and divided into two approximately equal portions. Each *S. canaria* was fed with one portion by directly placing the infected blood in the bird’s beak using a pipette.

Third, two *S. canaria* were exposed to the *Lankesterella* parasites by injection, using infected blood from *A. schoenobaenus* AS1 (Table 1). The freshly prepared mixture of infected blood, 3.7% solution of sodium citrate, and 0.9% saline (proportion of 4:1:5) was injected intramuscularly. About 100 µL of this mixture was inoculated in the pectoral muscle of the two recipient *S. canaria*. A group of intact *S. canaria* (7 birds), which were permanently maintained in the same vivarium, was used as controls in the second and third experiments; control testing aimed to prove the absence of the natural transmission of *Lankesterella* in the vivarium.

To check if the *S. canaria* were susceptible, blood was collected from the experimental birds every 3–4 DPE for almost 6 months (160 DPE). Blood collection was performed as described above, and each sample was examined using the buffy-coat method [22], microscopic examination of Giemsa-stained blood films [21], and PCR-based methods, as described below. Control birds remain non-infected throughout entire observation.

### 2.6. DNA Extraction, PCR and Sequencing

Blood samples, residual parts of the dissected insects and dead non-dissected experimental insects were examined for *Lankesterella* parasites using PCR amplification. Total DNA was extracted from all samples using a standard ammonium acetate protocol [25]. A nested PCR protocol was used to amplify a section of the *18S* ribosomal RNA gene. The primers used were designed based on 272 sequences of Apicomplexa parasites and vertebrates host (Supplementary Table S1). The first pair of primers Cocc18S_n1F (5′-CAGCTTTCGACGGTATGGTATTGG-3′) and Cocc18S_n1R (5′-CAGACCTGTTATTGCCTCAAACTTCCT-3′) amplify a fragment of 1135 bp. The second pair of primers Cocc18S_n2F (5′-GTATTGGCTTACCGTGGCAGTGAC-3′) and Cocc18S_n2R (5′-GCCTCAAACTTCCTTGCGTTAGACA-3) amplify a fragment of 1105 bp. We performed PCR amplification in 25 µL of total volume, including 50 ng of total genomic DNA template (2 µL), 12.5 µL of DreamTaq Master Mix (Thermo Fisher Scientific, Vilnius, Lithuania), 8.5 µL nuclease-free water, and 1 µL of each primer (10 µM concentration). One positive (infection confirmed by microscopic analysis) and one negative control (ultrapure water) were used on each run. The PCR started with an initial denaturation at 95 °C (2 min), followed by 35 cycles of 95 °C (30 s), 58 °C (30 s), 72 °C (1 min), and a final extension at 72 °C (5 min). Positive results were visualized by electrophoresis, applying 2 µL of the final PCR product on a 2% agarose gel. Amplicons of proper length (approximately 1105 bp) were precipitated and sequenced from both ends with primers Cocc18S_n2F/Cocc18S_n2R using Big Dye Terminator V3.1 Cycle Sequencing Kit and ABI PRISMTM 3100 capillary sequencing robot (Applied Biosystems, Foster City, CA, USA). The obtained sequences were edited and aligned to obtain a consensus sequence, using the BioEdit software version 7.0.5.3 [26]; they were then deposited in the GenBank database (accession numbers MW727631-MW727689).

### 2.7. Plylogenetic Analysis

A Bayesian phylogeny was constructed using MrBayes version 3.2.6 [27]; the alignment consisted of 78 sequences, including those obtained in this study. DNA sequences of Apicomplexa parasites belonging to 12 genera were used; they belonged to *Isospora*, *Eimeria*, *Hepatozoon*, *Schellackia*, *Goussia*, *Sarcocystis*, *Frenkelia*, *Hammondia*, *Toxoplasma*, *Hemolivia*, and *Haemogregarina*. Sequences of haemosporidian parasites (*Plasmodium*, *Haemoproteus* and *Leucocytozoon* species) were used as outgroup. The GenBank accession number of sequences used in the phylogenetic analysis are given in Supplementary Table S2.

Sequences were aligned using MAFFT software version 7 [28]. The General Time Reversible (GTR) was selected by the software MrModelTest2 software version 2.3 [29] as the best-fit substitution model. The run was conducted with four chains and with a sampling frequency of every 100th generation over 3 million generations. We discarded 25% of the trees as ‘burn-in’. The remaining trees were used to construct a consensus tree. The phylogenetic trees were visualized using FigTree version 1.4.0 software [30]. The sequence divergence between different lineages was calculated using the Jukes-Cantor model of substitution, with all substitutions weighted equally (uniform rates), implemented in the MEGA-X software version 10.1.8 [31].

### 2.8. Statistical Analysis

Descriptive statistical analyses for parasite morphometry were carried out using ‘R studio’ version 2.4.3 [32]. Mosquito survival statistical analyses were done using the software SPSS Statistics v. 22 (IBM Corp. Copyright) to calculate the Kaplan-Meier curve, and the reported survival curves of all groups were compared using the Log-Rank test [33]. Insects collected for dissection and experimental infections of *S. canaria* were considered censored [33]. The differences between curves were considered as significant when *p* < 0.05.

## 3. Results

### 3.1. Prevalence of Infection

In total, samples from 641 birds were processed. The overall prevalence of *Lankesterella* infection was 8.6% (Table 2). The samples of adult birds of 16 species belonging to six families of Passeriformes were examined. *Lankesterella* infections were observed in species of Acrocephalidae, Hirundinidae, and Paridae (Table 2). Only juveniles of Acrocephalidae species were examined, and three of four tested species were positive; *A. schoenobaenus* was the most prevalently infected species, in both adults and juveniles (Table 2). These are the first reports of *Lankesterella* parasites in all studied bird species, except for *A. schoenobaenus* and great tits *Parus major*.

### 3.2. Molecular and Morphological Characterization

**Figure 1:**
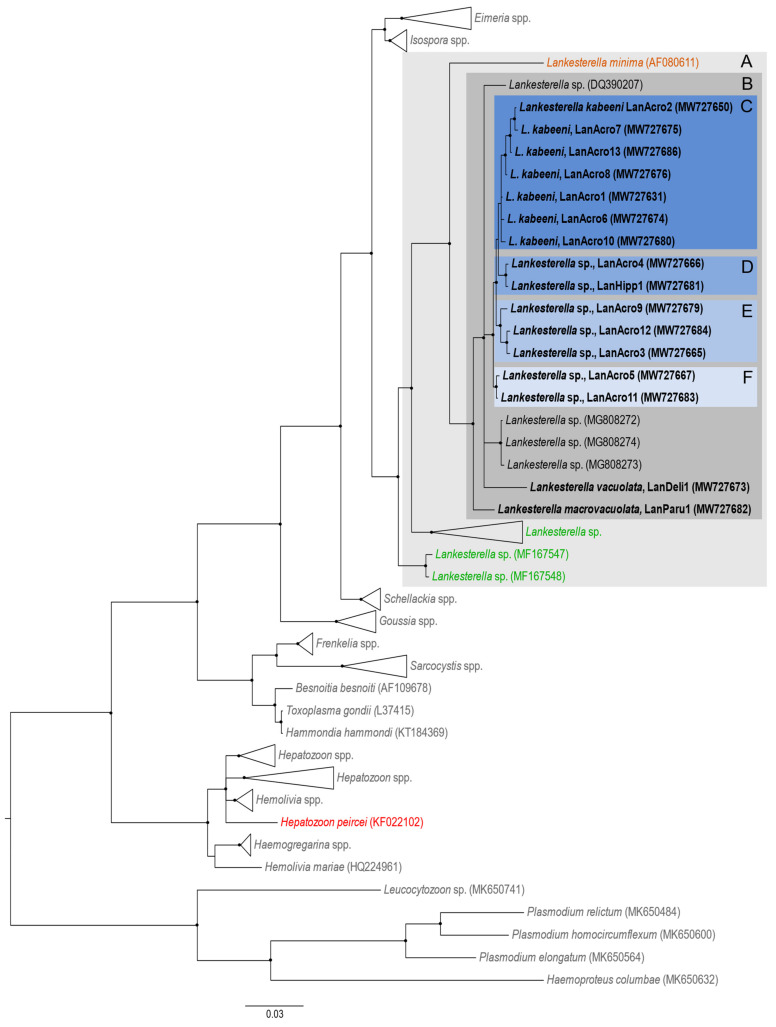
Bayesian inference tree of partial *18S* rRNA gene sequences of *Lankesterella* parasites. Haemosporidian parasite DNA sequences (*Plasmodium* and *Haemoproteus*) were used as outgroup. *Lankesterella* DNA sequences from birds (given in black), amphibians (in orange) and reptiles (in green) were placed in a well-supported clade (A) and they are phylogenetically related to *Isospora* and *Eimeria*. Sequences obtained in this study are given in bold. Nodes with posterior probability of ≥ 85% were marked with black dots. Note that sequences of avian *Lankesterella* clustered in one well-supported clade (B); lineages from sedge warblers *Acrocephalus schoenobaenus* were placed in cluster (C); some lineages were shared between marsh warblers *A. palustris* and Eurasian reed warblers *A. scirpaceus*, and they grouped together in clade (D), but other ones clustered with lineages from great reed warbler *A. arundinaceus* (see clade F); one *Lankesterella* lineages from *A. scirpaceus* clustered with the lineage recorded from the icterine warblers *Hippolaris icterina* (E). All avian *Lankesterella* lineages were closely related to *Lankesterella minima*, which is the genus type species parasitizing frogs. It should be noted that *Hepatozoon piercei* (in red) appeared in a clade distant from the *Lankesterella* sequences.

In all, 16 different *Lankesterella* lineages were identified. They were named using the prefix “Lan” followed by the first four letters of the host genus and a sequential number (LanAcro1 to LanAcro13, LanDeli1, LanParu1, LanHipp1). They all grouped in one well-supported clade during phylogenetic analysis (Figure 1). All *Lankesterella* sequences from birds, lizards, and amphibians clustered together in one well-supported clade (Figure 1, clade A), indicating a close phylogenetic relationship of the parasites inhabiting different groups of vertebrates. Interestingly, all lineages of avian parasites detected in this study were closer related to *Lankesterella minima*, the parasite of amphibians, than to sequences reported in lizards (Figure 1). All *18S* sequences obtained from birds clustered together (Figure 1, clade B). Different bird species were infected by different *Lankesterella* lineages, with the exception to the marsh warblers *Acrocephalus palustris* and the Eurasian reed warblers *Acrocephalus scirpaceus* (Table 3).

The highest diversity of lineages (seven) was found in *A. schoenobaenus* (Table 3; Figure 1, clade C). The *Lankesterella* lineage LanAcro4 from *A. scirpaceus* clustered with the lineage LanHipp1 recorded in the icterine warbler *Hippolais icterina* (Figure 1, clade D). One lineage shared by *A. palustris* and *A. scirpaceus* (LanAcro5) clustered with a lineage found only in *A. palustris* (Figure 1, clade F). The only lineage found in the great reed warbler *Acrocephalus arundinaceus* (LanAcro9) clustered with sequences obtained from *A. palustris* and *A. scirpaceus* (Figure 1, clade E).

Some lineages were found exclusively in juveniles (LanAcro3 and LanAcro4), while others were shared between juvenile and adult birds (LanAcro1, LanAcro2, and LanAcro5) (Table 3).

The lineages found in the great tits *Parus major* (LanParu1) and the common house martins *Delichon urbicum* (LanDeli1) were placed in a sister clade of the DNA sequences found in Acrocephalidae birds (Figure 1, clade B). These two parasites are readily distinguishable new species (see description below).

It is important to note that we did not detect any *Hepatozoon* sequences in the tested samples, however, the used primers also amplified *Isospora* and *Caryospora* parasite DNA (results not shown).

**Figure 2:**
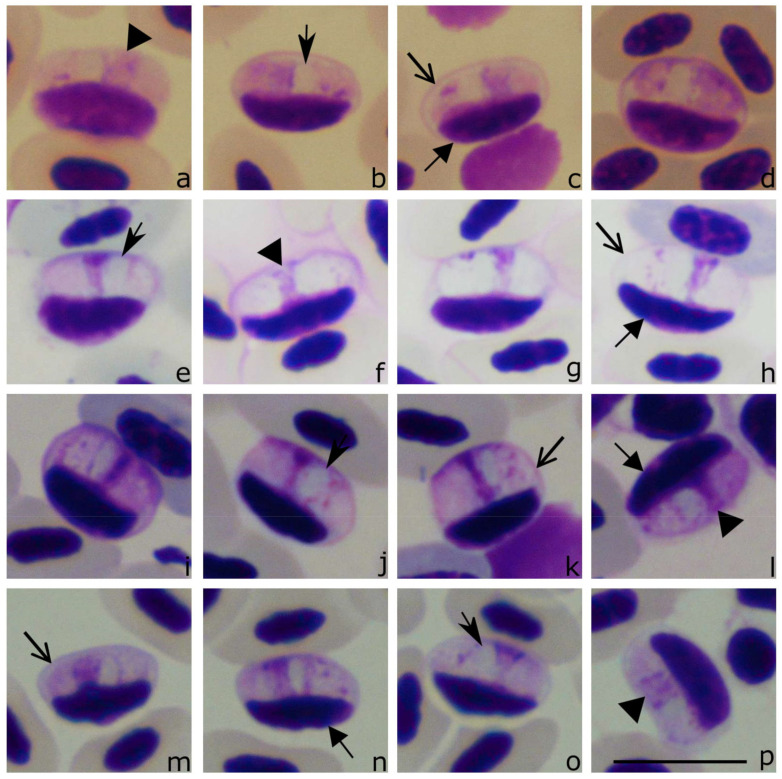
Sporozoites (**a**–**p**) of *Lankesterella* sp. *18S* rRNA lineages: LanAcro4 from the Eurasian reed warblers *Acrocephalus scirpaceus* (**a**–**d**) (see clade D in Figure 1), LanHipp1 from the icterine warbler *Hippolais icterina* (**e**–**h**) (clade D in Figure 1), LanAcro9 from the great reed warbler *Acrocephalus arundinaceus* (**i**–**l**) (clade E, Figure 1), and LanAcro5 from the Eurasian reed warblers *Acrocephalus scirpaceus* (**m**–**o**) (clade F, Figure 1). Note that all sporozoites develop in thrombocytes, whose relatively thick cell envelope is readily visible (**c**,**h**,**k**,**m**). The parasite nucleus is elongate and occupies entire parasite width (**a**,**f**,**l**,**p**); markedly condensed chromatin is sometimes visible (**l**). A vacuole-like structure is closely appressed to the parasite nucleus (**b**,**e**,**j**,**o**). Triangle, parasite nucleus. Barbed arrowhead, vacuole-like structure. Short triangle arrow, host cell nucleus. Simple wide arrowheads, thrombocyte envelope. Scale bar: 10 µm. Methanol fixed and Giemsa-stained blood films.

**Figure 3:**
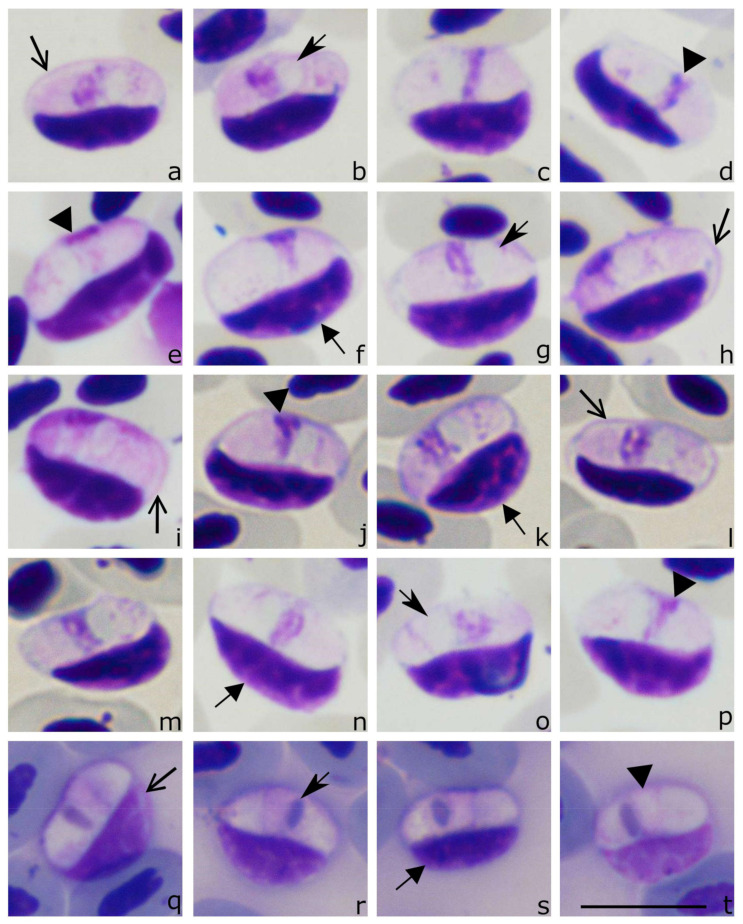
Sporozoites (**a**–**t**) of *Lankesterella kabeeni 18S* rRNA lineage LanAcro1 from the sedge warbler *Acrocephalus schoenobaenus*: images from neoparahapantotype (**a**–**p**) and hapantotype (**q**–**t**, unknown lineage) are shown. Parasites develop in thrombocytes, whose host cell identity is readily distinguishable due to non-stained host cell cytoplasm and relatively thick host cell envelope (**a**,**h**,**i**). Sporozoites are closely appressed to the host cell nuclei, which are displaced to the envelope of host cells (**f**,**d**,**l**). Note the presence of a vacuole-like structure, which is closely appressed to the parasite nucleus (**b**,**g**,**j**). The parasite nucleus is of band-like shape and occupies the entire width of the sporozoite (**d**,**e**,**j**). Triangle, parasite nucleus. Short, barbed arrow, vacuole-like structure. Short triangle arrow, host cell nucleus. Simple wide arrow, host cell envelope. Scale bar: 10 µm. Methanol fixed and Giemsa-stained (**a**–**p**) and Haemacolor-stained blood films (**q**–**t**).

**Figure 4:**
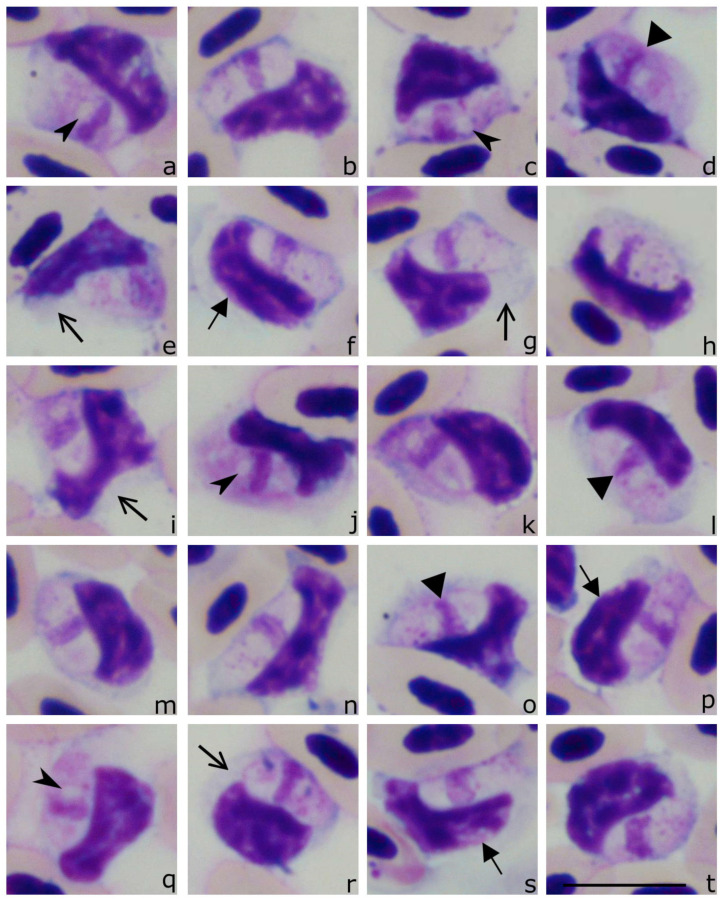
Sporozoites (**a**–**t**) of *Lankesterella vacuolata* n. sp. *18S* rRNA lineage LanDeli1 from the common house martin *Delichon urbicum*. Parasites develop in mononuclear leukocytes, which are readily distinguishable from other leukocytes due to basophilic staining of the cell cytoplasm (**e**,**g**,**i**,**r**). Note that sporozoites are closely appressed to the host cell nuclei, which frequently assume comma-like shapes (**b**,**f**,**i**,**m**,**n**,**p**). A vacuole-like structure is small, does not adhere to the parasite nucleus (**a**,**c**,**j**,**q**); the latter is elongate and occupies the entire width of the sporozoite (**d**,**l**,**o**). Triangle, parasite nucleus. Barbed arrow, vacuole-like structure. Short triangle arrow, host cell nucleus. Simple wide arrow, host cell cytoplasm. Scale bar: 10 µm. Methanol fixed and Giemsa-stained blood films.

**Figure 5:**
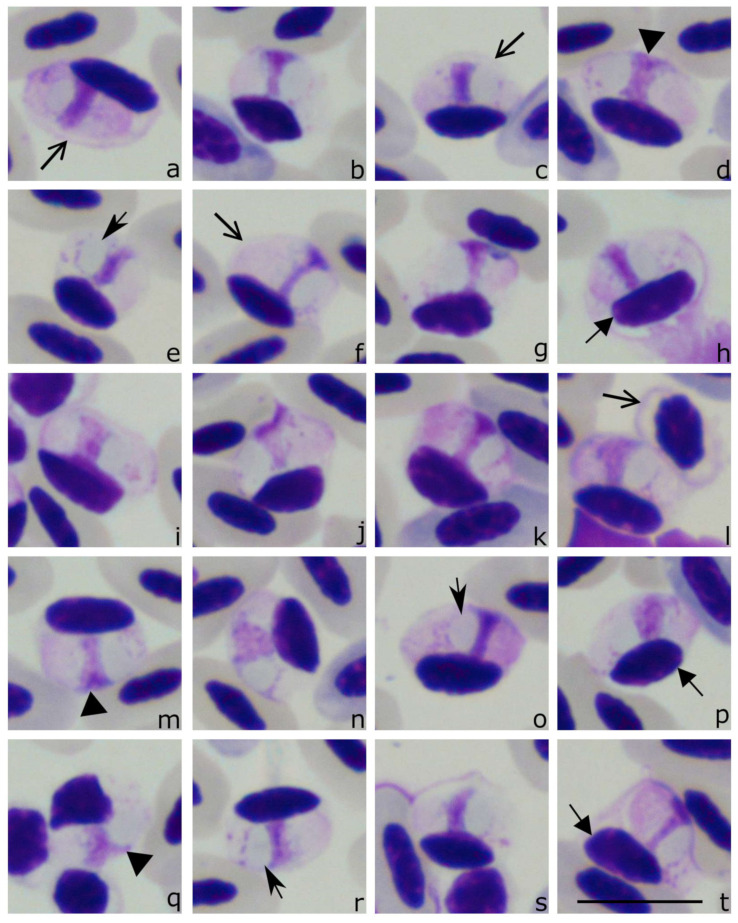
Sporozoites (**a**–**t**) of *Lankesterella macrovacuolata* n. sp. *18S* rRNA lineage LanParu1 from the great tit *Parus major*. The parasites develop in thrombocytes (**l**), in which host cell identity is readily distinguishable due to the non-stained cytoplasm (**a**,**c**,**f**) and relatively thick cell envelope (**a**,**f**). Note that the sporozoites are closely appressed to the host cell nuclei, which are displaced to the envelope of the host cells (**b**,**e**,**h**,**p**,**r**). The presence of a big vacuole-like structure, which is closely appressed to the parasite nucleus (**e**,**o**,**r**) is an important species characteristic. Parasite nucleus is of band-like shape; it occupies the entire width of the sporozoite (**c**,**d**,**f**,**h**,**j**). Triangle, parasite nucleus. Short, barbed arrow, vacuole-like structure. Short triangle arrow, host cell nucleus. Simple wide arrow, host cell envelope. Scale bar: 10 µm. Methanol fixed and Giemsa-stained blood films.

The following general morphological features of *Lankesterella* sporozoites are worth mentioning. Blood stages of *Lankesterella* parasites (sporozoites) were observed in individuals belonging to six of seven PCR positive bird species (Table 2, Figure 2, Figure 3, Figure 4 and Figure 5). Some of them inhabited leukocytes (Figure 4) while others were seen in thrombocytes (Figure 2, Figure 3 and Figure 5). In both types of host cells, intracellular parasites assumed bean-like shapes (Figure 2, Figure 3, Figure 4 and Figure 5). However, when *Lankesterella* sporozoites infect leukocytes, they induce a bigger deformation of the host-cells (compare Figure 2 with Figure 4).

The parasite nucleus was not always equally well-visible in all preparations (compare Figure 2b,d–h); this depends on blood film intensity staining. When nuclei were well-visible, their shape varied, and it was often band-like (see Figure 2k–n and Figure 3c,d) or triangle-like (Figure 2h–o). Regardless of the form, the nuclei were usually located centrally or slightly sub-centrally in sporozoites of all lineages. A vacuole-like structure was present in all examined parasites; it was usually closely appressed to the parasite nucleus (Figure 2, Figure 3, Figure 4 and Figure 5).

### 3.3. Redescription of Lankesterella kabeeni and Description of New Lankesterella Parasites

*Lankesterella kabeeni* Kruszewicz and Drycz, 2000 new combination (Figure 3, Table 4).

Synonym: *Hepatozoon kabeeni* Kruszewicz and Drycz, 2000.

Type host: the sedge warbler *Acrocephalus schoenobaenus* Linnaeus, 1758 (Passeriformes, Acrocephalidae).

Additional hosts: unknown.

Type locality: Hapantotype originated from a site near Biebrza river, Northeast Poland and Milicz fishponds, Southeast Poland. Our study was based on material collected at the Ornithological Station, Ventės Ragas, Lithuania (55°20′28.1″ N 21°11′25.3″ E).

Site of infection: Thrombocytes; no other data.

Prevalence: 17.7% (41 out of 232 examined *A. schoenobaenus* were infected).

Type specimens: Hapantotype (blood films were stored at Warzaw Zoological Garden, Ratuszowa 1/3, 03-461 Warszawa, Poland). Neoparahapantotype: blood film (collection number 49287NS, adult *Acrocephalus schoenobaenus* with parasitaemia of 1% or 1 parasite per 100 thrombocytes, Giemsa stained, Ornithological Station, Ventės Ragas, collected 05.18.2018 by M. Ilgūnas) was deposited in the Nature Research Centre, Vilnius, Lithuania. *Lankesterella* parasites were marked with circles in neoparahapantotype preparation.

DNA sequences: *18S* ribosomal RNA gene lineage LanAcro1 (1030 bp, GenBank accession number MW727631). This is the most prevalent lineage of the parasite in the type avian host; it was found in 8.6% of examined type avian hosts. Other lineages were LanAcro2 (MW727650), LanAcro6 (MW727674), LanAcro7 (MW727675), LanAcro8 (MW727676), LanAcro10 (MW727680), and LanAcro13 (MW727686).

Distribution: this parasite has been reported only in the type hosts and some other species of the Acrocephalidae (Table 2 and Table 3). All of these birds winter is in sub-Saharan Africa. So far, this parasite has been reported in Poland (original description) and in Lithuania (this study). There is no DNA sequence with 100% similarity in GenBank.

Vector: unknown.

ZooBank registration: the life science identifier (LSID) for the new combination *L. kabeeni* is urn:lsid:zoobank.org:pub:7DAEB1BB-96F1-470F-85DC-5392EF671FC9.

Etymology: the species name was given to thank the Polish Committee for scientific research (in Polish Komitet Badań Naukowych—KBN) for financially supporting the original study, which discovered this parasite in Poland.

Sporozoites (Figure 3a–t, Table 4) are elongate, with equally rounded ends; the parasites occupy the entire cytoplasm of the host cell (Figure 3m–o). The sporozoite cytoplasm stains pale-reddish, heterogeneous in appearance. The outline is even. The sporozoites are closely appressed to the infected cell nuclei, which are displaced towards the cell envelope (Figure 3f,k,n,s). The sporozoites are slightly bigger than the nuclei of the infected cell (Figure 3b,k,q). The sporozoite nuclei are usually elongate and band-like with a markedly condensed chromatin (Figure 3d,e,j,t); they assume a central or slightly subcentral position and usually occupy the entire width in sporozoites (Figure 3c,d,m,q). A roundish to elongated (Figure 3a,b,g) vacuole-like structure is present in each sporozoite (Table 4); it is usually closely appressed to the parasite nucleus and can be pale-stained when treated with Giemsa (Figure 3a,b,g) or purple when stained with Haemacolor (Figure 3q–t). This should be taken into consideration regarding the identification of *Lankesterella* parasites.

**Table 4:** Morphometry of sporozoites of *Lankesterella kabeeni* (*18S* rRNA lineage LanAcro1), *Lankesterella vacuolata* n. sp. (LanDeli1), and *Lankesterella macrovacuolata* n. sp. (LanParu1).

Feature	*Lankesterella kabeeni* (*n* = 21) ^a^	*Lankesterella vacuolata* (*n* = 21)	*Lankesterella macrovacuolata* (*n* = 21)
Parasite			
Length	8.2–9.9 (9.1 ± 0.4) ^b^	7.4–9.9 (8.2 ± 0.7) ^b^	8.2–10.6 (9.1 ± 0.6)
Width	2.6–4.0 (3.4 ± 0.4)	2.7–4.8 (3.6 ± 0.5)	3.2–4.7 (3.9 ± 0.4)
Area	21.9–29.3 (25.7 ± 2.2)	19.8–39.4 (25.0 ± 4.1)	24.7–33.2 (29.3 ± 2.2)
Parasite nucleus			
Length	1.0–2.5 (2.0 ± 0.4)	1.2–2.3 (1.6 ± 0.3)	1.2–4.0 (2.4 ± 0.7)
Width	1.5–3.6 (2.7 ± 0.6)	2.7–3.8 (3.2 ± 0.3)	2.7–4.4 (3.6 ± 0.4)
Area	2.7–5.4 (3.9 ± 0.7)	3.4–5.6 (4.4 ± 0.6)	4.1–8.1 (5.8 ± 1.3)
Parasite vacuole-like structure			
Length	1.0–2.0 (1.5 ± 0.2)	0.7–1.6 (1.0 ± 0.2)	1.3–3.0 (1.9 ± 0.4)
Width	1.4–2.9 (2.2 ± 0.4)	1.2–2.3 (1.5 ± 0.3)	2.2–3.7 (2.9 ± 0.4)
Area	1.2–3.8 (2.8 ± 0.7)	0.9–2.3 (1.3 ± 0.4)	3.5–7.2 (4.6 ± 0.8)

^a^ Number of measured parasites. ^b^ All measurements are given in micrometers. Minimum and maximum values are provided, followed in parentheses by the arithmetic mean and standard deviation (SD).

Taxonomic remarks: other than that mentioned in the original description, this parasite infects thrombocytes, not leukocytes. This feature was visible in both the hapantotype and neoparahapantotypes. The origin of the thrombocytes can be readily identified due to the presence of a colorless cytoplasm and the readily distinguishable outline of the host cell envelope (Figure 3a,h,i,l,q), which is not the case in other avian blood cells [34]. Moreover, a comparison of the data from neoparahapantotypes (Figure 3a–p, Table 4) with the original description (hapantotype, see [13]) shows some differences in the size of parasite nuclei and coloration of the vacuole-like structures (compare Figure 3a–p with Figure 3q–t); the latter are better visible in the hapantotype. This difference is mainly due to the different staining: the hapantotype was stained with Haemacolor and neoparahapantotypes (the present study) with Giemsa. The former stain better differentiates these cell structures and makes them more visible.

*Lankesterella kabeeni* is morphologically similar to *Lankesterella macrovacuolata* n. sp. (Figure 4, see description below). Sporozoites of these parasites can be distinguished by the following features. First, the average area of the vacuole-like structure in *L. kabeeni* is smaller (2.8 µm^2^) than in *L. macrovacuolata* n. sp. (4.6 µm^2^). Second, the average area of the nucleus in *L. kabeeni* sporozoites is smaller (3.9 µm^2^) than in *L. macrovacuolata* (5.8 µm^2^). The genetic difference between the partial *18S* rRNA gene fragments in these parasites is 22 bp or 2.2% (uncorrected p-distance).

*Hepatozoon sylviae* was reported infecting Acrocephalidae birds [15,16]. Due to morphological similarity, this parasite likely belongs to *Lankesterella*, and it can be distinguished from *L. kabeeni* due to the following features. First, the average parasite nucleus area is bigger in *H. sylviae* (5.9 µm^2^, see Bennett et al. [12]) than in *L. kabeeni* (3.9 µm^2^). Second, the average area of the vacuole-like structure is smaller in *H. sylviae* (1.2 µm^2^) than in *L. kabeeni* (2.8 µm^2^). A molecular characterization of *Hepatozoon sylviae* is absent.

*Lankesterella vacuolata* n. sp. (Figure 4, Table 4).

Type host: the common house martin *Delichon urbicum* Linnaeus, 1758 (Passeriformes, Hirundinidae).

Additional hosts: unknown.

Type locality: Ventės Ragas Ornithological Station, Lithuania (55°20′28.1″ N 21°11′25.3″ E)

Site of infection: mononuclear leukocytes; no other data.

Prevalence: 2.3% (1 out of 43 examined *D. urbicum* were infected).

Type specimens: Hapantotype (blood film collection number 49285NS, adult *Delichon urbicum* with parasitemia of 24% or 24 parasites per 100 mononuclear leukocytes, Giemsa stained, Ventės Ragas Ornithological Station, collected 05.18.2015 by M. Ilgūnas) was deposited in the Nature Research Centre, Vilnius, Lithuania; parahapantotype was deposited in the Queensland Museum, Brisbane, Australia (collection number G466228, other data as above). *Lankesterella* parasites were marked with circles in type preparations. A co-infection with *Haemoproteus* sp. and *Plasmodium* sp. is present in the type material.

DNA sequences: *18S* ribosomal RNA gene lineage LanDeli1 (1030 bp, GenBank accession number MW727673).

Distribution: this infection has been reported only in the type hosts and type locality as far. There is no sequence of 100% similarity deposited in GenBank.

Vector: unknown.

ZooBank registration: the LSID for *L. vacuolata* is urn:lsid:zoobank.org:act:9F25425D-650F-4DE4-BE64-BD34AD82EE7B.

Etymology: the species name refers to the presence of a small vacuole-like structure in the cytoplasm of sporozoites (Figure 4a,c,j,q), which is a characteristic feature of this parasite.

Sporozoites (Figure 4a–t, Table 4) develop in mononuclear leukocytes. The parasites are elongate, with approximately equally rounded ends (Figure 4h,k,m); they do not occupy the entire cytoplasm of host cells, and the cytoplasm remnants are well-visible in all infected cells (Figure 4a–t). The sporozoite cytoplasm stains pale reddish, heterogeneous in appearance. The outline is even. The sporozoites are closely appressed to the infected cell nuclei, which are slightly displaced towards the cell envelope; host-cell nuclei are deformed and frequently assume a comma-like shape (Figure 4f,l–o,s). The sporozoites are of similar length compared to the nuclei of the infected cells. The parasite nuclei are elongate, band-like in form; they assume a central or slightly subcentral position, and usually occupy the entire width of the sporozoites (Figure 4d,l,o). A roundish (Figure 4a,c) or slightly oval (Figure 4q) vacuole-like structure is present in each sporozoite; it is relatively small (Table 4), pale-stained, locates close to the parasite nucleus, but does not touch it, a characteristic feature of this species (Figure 4a,c–e,j). Sporozoites deform infected host cells, which look to be fragile, resulting in numerous variously deformed host-parasite complexes present in blood films (Figure 4).

Taxonomic remarks: the origin of the host cells (mononuclear leukocytes) can be readily identified due to the basophilic cytoplasm (Figure 4e,g,i,r), which is not the case in other avian blood cells [34]. The most distinctive morphological features of *Lankesterella vacuolata* n. sp. are the presence of a small vacuole-like structure located close to the parasite nucleus (Figure 4a,c,q) and the pale-reddish staining of the cytoplasm. Additionally, the host cells are relatively fragile, resulting in their deformation in blood films. Moreover, the nuclei of the host cells often assume a comma-like shape, but the latter feature has also been reported in *Hepatozoon sylvae*, *H. atticorae*, and *H. parus* [12]. Blood stages of *L. vacuolata* n. sp. can be distinguished from these parasites due to the following features. First, average area of nuclei in *L. vacuolata* sporozoites (4.4 µm^2^) is smaller than in *H. sylvae* (5.9 µm^2^). Second, the average area of *L. vacuolata* sporozoites is smaller (25 µm^2^) than in *H. parus* (34 µm^2^) and *H. atticorae* (29.6 µm^2^) [12]. Third, the area of the vacuole-like structure is bigger in *H. parus* (3.1 µm^2^) than in L. *vacuolata* (1.3 µm^2^).

*Lankesterella macrovacuolata* n. sp. (Figure 5, Table 4).

Type-host: the great tit *Parus major* Linnaeus, 1758 (Passeriformes, Paridae).

Additional hosts: unknown.

Type locality: Ventės Ragas Ornithological Station, Lithuania (55°20′28.1″ N 21°11′25.3″ E)

Site of infection: Thrombocytes; no other data.

Prevalence: 16.7% (1 out of 6 examined *P. major* were infected).

Type-specimens: Hapantotype (blood film collection number 49286NS, adult *Parus major* with parasitaemia of 5% or 5 parasites per 100 thrombocytes, Giemsa stained, Ventės Ragas Ornithological Station, collected 05.16.2018 by M. Ilgūnas) was deposited in the Nature Research Centre, Vilnius, Lithuania; parahapantotype was deposited in the Queensland Museum, Brisbane, Australia (collection number G466229, other data as above). *Lankesterella* parasites were marked with circles in type preparations. A co-infection with *Leucocytozoon* sp., and *Haemoproteus* sp. is present in the type material.

DNA sequences: *18S* ribosomal RNA gene lineage LanParu1 (1030 bp, GenBank accession number MW727682).

Distribution: this parasite has only been found in the great tit. There is no sequence of 100% similarity deposited in GenBank.

Vector: unknown.

ZooBank registration: the LSID for *L. macrovacuolata* is urn:lsid:zoobank.org:act:8A29B18F-5D3F-48D4-9FB2-606F2B92C3A7.

Etymology: the species name refers to the presence of a big vacuole-like structure in the cytoplasm of the sporozoites, which is a characteristic feature of this parasite.

Sporozoites (Figure 5a–t, Table 4): infect thrombocytes. The sporozoites are elongate, with equally rounded ends; fully grown parasites occupy the entire cytoplasm of the host cell (Figure 5a–t). The sporozoite cytoplasm stains pale-reddish, heterogeneous in appearance. The outline is even. The sporozoites are closely appressed to the infected cell nuclei, which are displaced towards the cell envelope (Figure 5f,l–o,s). The sporozoites are bigger than the nuclei of infected cell and they often slightly enclose the nuclei (Figure 5d,i,o). The sporozoite nuclei usually are elongate, band-like with markedly condensed chromatin (Figure 5e); the nuclei assume a central or subcentral position and usually occupy the entire width of the sporozoites (Figure 5d,q). A roundish (Figure 5e,o,r) vacuole-like structure is present in each sporozoite; it is relatively big (Table 4), pale-stained, and closely appressed to the parasite nucleus (Figure 5a,e,k,l,o). The sporozoites deform and enlarge infected cells (for comparison, see Figure 5l, which shows infected and uninfected thrombocyte in same image).

Taxonomic remarks: the origin of host cells (thrombocytes) can be readily identified due to the presence of a colorless cytoplasm and a clear cell envelope (Figure 5a,c,f), which is not the case with other avian blood cells [34].

The most distinctive morphological feature of *Lankesterella macrovacuolata* n. sp. is the presence of a big vacuole-like structure in the sporozoites (Figure 5a,e,k,l,o). This structure is bigger than other homologous structures seen in all described avian *Hepatozoon* species and *Lankesterella* species that were found during this study. Even though the average area of these structures is similar to that which was reported in *Hepatozoon nephrontis* (3.9 µm^2^, see Bennett et al. [12]), in *L. macrovacuolata* n. sp. it is still bigger (4.6 µm, Table 4). These two species can be distinguished from each other by the location of the parasites in the infected cell. *Hepatozoon nephrontis* can be seen enclosed by the infected cell nucleus, forming a “cyst-like wall” that surrounds the parasite [12]. This is not the case in *L. macrovacuolata* n. sp. Blood stages of *L. macrovacuolata* n. sp. can be distinguished from *H. parus*, a species reported in the same avian host species, by its bigger vacuole-like structure, whose average areas are 4.6 µm^2^ and 3.1 µm^2^, respectively. Among other features which are helpful to distinguish between these two parasites, the origin of the host cells should be mentioned. Mainly, *L. macrovacuolata* infects thrombocytes and *H. parus* was reported infecting mononuclear leukocytes. The influence of the parasites on the host cell nuclei is also different. In *H. parus*, the nuclei can assume U-like shapes and enclose the parasite [35], but this is not a case in *L. macrovacuolata* (Figure 5).

### 3.4. In Vitro Development of Lankesterella kabeeni

**Figure 6:**
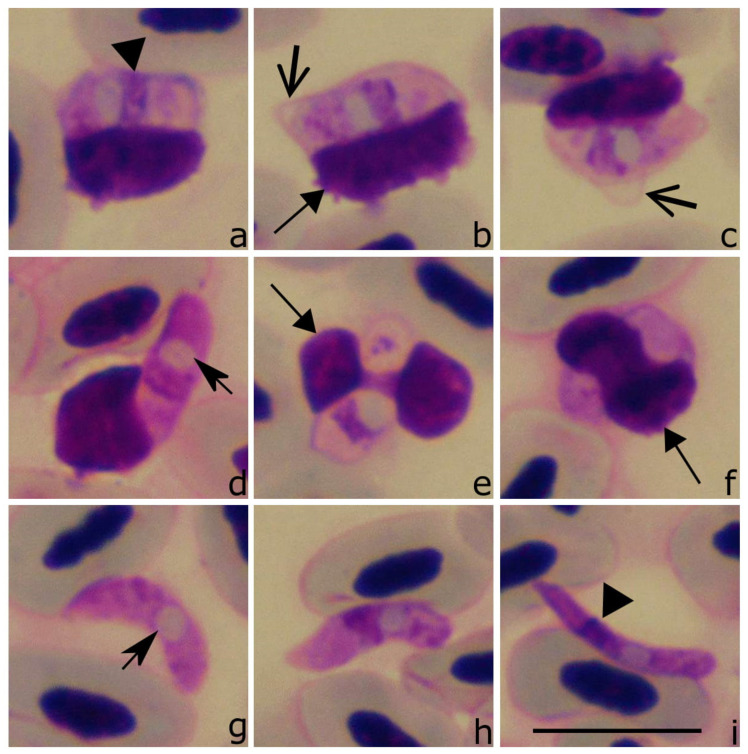
In vitro sporozoites (**a**–**i**) of *Lankesterella kabeeni 18S* rRNA lineage LanAcro8 isolated from the sedge warbler *Acrocephalus schoenobaenus*. Sporozoite in a circulating thrombocyte before exposure blood to air (EBA) (**a**). Note that the sporozoites deform the host cell envelope 1 min after EBA (**b**,**c**), when they start to escape from host cells (**d**). Within 3 min after EBA, the deformation of infected host cells is readily visible during this process (**e**,**f**). Free (extracellular) sporozoites were frequently seen 1h after EBA (**g**–**i**). Triangle, parasite nucleus. Short, barbed arrow, vacuole-like structure. Short triangle arrow, host cell nucleus. Simple wide arrow, host cell envelope. Scale bar: 10 µm. Methanol fixed and Giemsa-stained blood films.

*Lankesterella kabeeni* sporozoites escaped from host cells very rapidly. Within 1 min after the EBA, sporozoites were seen at the stage of initiation of escape from infected cells (Figure 6d), but the majority were still intracellular (Figure 6a–c). At this stage of development, a slight deformation of the host cell envelope was visible (Figure 6b–c); this feature was not observed in blood films prepared using fresh blood, which was not exposed to air (compare Figure 1, Figure 2, Figure 3 and Figure 4 with Figure 6b–c) thus, the host cell envelope changes were certainly induced by escaping sporozoites. Within 3 min after EBA, free sporozoites were more frequently seen, and the parasites deforming the host cells and their nuclei were numerous (Figure 6e–f). The number of extracellular sporozoites gradually increased, and intracellular parasites were extremely rare 1 h after EBA, and they were not observed 2 h and later after EBA. It is worth noting that two different forms of sporozoites were seen. First, the broad-width forms possessing nuclei with relatively low chromatin condensation (Figure 6g,h). Second, narrow-width forms possessing nuclei with a highly condensed chromatin (Figure 6i). The presence of these two different sporozoite forms was not related to the time passed after the EBA; they were usually seen simultaneously.

### 3.5. Development of Lankesterella kabeeni in Experimentally Infected Mosquitoes and the Life Span of Infected Mosquitoes

**Figure 7:**
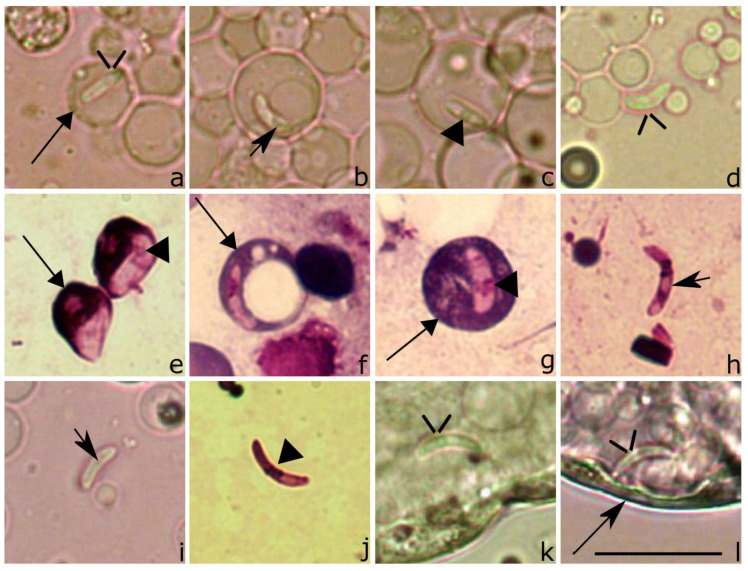
Sporozoites of *Lankesterella kabeeni 18S* rRNA lineages LanAcro1 (**a**,**b**,**d**) and LanAcro8 (**c**,**e**–**l**) in experimentally infected *Aedes aegypti* after blood meal on the naturally infected sedge warblers *Acrocephalus schoenobaenus*. The images show sporozoites in wet preparation (**a**–**d**,**i**,**k**,**l**) and methanol-fixed and Giemsa-stained preparation (**e**–**h**,**j**). Sporozoites inside mosquito haemocytes (**a**–**c**,**e**–**g**); they were also seen located free after the midgut was crashed (**d**,**h**–**j**) and inside mosquito midgut (**k**,**l**). Triangle, parasite nucleus. Short, barbed arrow, vacuole-like structure. Simple wide arrowheads, sporozoites. Long barbed arrow, basal lamina. Long triangle arrow, haemocytes. Scale bar: 10 µm.

Overall, 114 mosquitoes were fed on parasite donor birds (86 *Ae. aegypti* and 28 *Cx. p.* forma *molestus*); 68 mosquitoes were fed on negative control birds (36 *Ae. aegypti* and 32 *Cx. p.* forma *molestus*) (Table 2). Microscopic examination showed the presence of *L. kabeeni* sporozoites in all infected and dissected mosquitoes, but they were absent in control insects. In infected mosquitoes (in vivo), one end was more pointed than the other end (Figure 7d) in all extracellular sporozoites, similar to that which was seen in the in vitro experiment (Figure 6h,i). In intact isolated midguts, the parasites were frequently observed close to the basal lamina and between cells in the midgut epithelium (Figure 7k–l). In both cases, it was possible to see that sporozoites were alive and moving between the epithelial cells even until 42 DPE, indicating the long-lasting parasite viability in vectors. After the infected midgut was smashed and examined at high magnification of the microscope, the *Lankesterella* sporozoites were also seen inside haemocytes (Figure 7a–c,e–g) and often observed extracellularly (Figure 7d,h–j). In the latter case, the parasites were seen slowly moving; the movement was due to a slow flexing of the pointed anterior portion of the cell, followed by flexing the opposite cell side and resulting motion forward. We did not observe the presence of sporozoites in the mosquito head or thorax preparations; the midgut was the only site in which sporozoites were located. PCR-tested mosquitoes were positive for *L. kabeeni*, and the sequences obtained from these mosquitoes were the same as in the donor birds.

In the experimentally infected mosquitoes, the sporozoites were the only observed life cycle stage; they were observed in both exposed mosquito species. Alive *L. kabeeni* sporozoites, which were able to move, persisted in mosquito midguts for a long period; they were seen 42 DPE in *Ae. aegypti* and 21 DPE in *Cx. p.* forma *molestus*.

The average mosquito life span varied after exposure to different *Lankesterella* lineages and in different mosquito species. After exposure to LanAcro8 lineage, *Ae. aegypti* survived longer (on average 29.2 days) than *Cx. p.* forma *molestus* (on average 19.2 days) (*p* < 0.05). *Aedes aegypti* mosquitoes infected with the LanAcro8 lineage lived longer (on average 29.2 days) than the same insect species infected with the LanAcro1 lineage (on average 7.2 days) (*p* < 0.05). Interestingly, *Ae. aegypti* infected with LanAcro8 also survived longer (on average 29.2 days) than non-infected insects (on average 11.5 days) (*p* < 0.05). However, the life span was shorter (on average 7.2 days) when *Ae. aegypti* were infected with parasites of the lineage LanAcro1 (*p* < 0.05). Exposed *Cx. p.* forma *molestus* mosquitoes infected with LanAcro8 survived longer (on average 19.2 days) than the controls (on average 8.7 days), but this difference was insignificant (*p* > 0.05).

### 3.6. Experimental Infections of S. canaria

All attempts to experimentally infect *S. canaria* with the *L. kabeeni* lineage LanAcro8 from *A. schoenobaenus* were unsuccessful. All blood samples collected from the experimentally exposed *S. canaria* were negative after application of diagnostic techniques (microscopic analysis, buffy coat method, and PCR-based testing).

## 4. Discussion

The key results of this study are (i) the proof that *Lankesterella* species are present in many species of passerine birds and are phylogenetically related to other *Lankesterella* parasites of birds, lizards, and amphibians (Table 2, Figure 1); (ii) the confirmation that intracellular elongate apicomplexan parasites, which inhabit thrombocytes and leukocytes of birds, belong to *Lankesterella*, but not *Hepatozoon* in the tested samples; (iii) the discovery of 16 new closely related lineages of avian *Lankesterella* parasites and the development of molecular and morphological characterization of some of them; (iv) the description of two new *Lankesterella* species (i.e., *L. vacuolata* n. sp. and *L. macrovacuolata* n. sp.) and re-description of *L. kabeeni* (synonym *H. kabeeni*); (v) the description of the in vitro and in vivo development of *L. kabeeni* and the report of the long-time persistence of its sporozoites in mosquitoes, and (vi) experimental and molecular evidence of high vertebrate-host specificity of these avian parasites. These findings are discussed below in more detail.

This study showed a relatively low prevalence of *Lankesterella* parasites in the examined passerine birds; the overall infection prevalence was 8.6%; it was similar and reached 9.7% and 6.9% in adults and juveniles, respectively. Information about the prevalence of *Lankesterella* infections in wild birds is limited, thus it is difficult to compare the prevalent data of this study and the corresponding information from the literature. In *A. schoenobaenus*, the prevalence of infections in adults was high (33.3%, Table 2), which is still lower than the same bird species in Poland (47.3%) [5]. Interestingly, 31.2% of the Eurasian blue tits, *Cyanistes caeruleus*, were positive for *Lankesterella* infections in a study conducted in Spain [4], and this is close to the prevalence in *A. schoenobaenus*, investigated in the present study. However, if only *A. schoenobaenus* juveniles are considered, the prevalence of infection in Lithuania was less (8.1%) than reported in the juvenile snow bunting *Plectrophenax nivalis* (20%) in Norway [20]. These differences might be due to various biotic and abiotic factors influencing the transmission, particularly susceptibility of bird species or/and epidemiological situation at study sites. To answer these questions, more studies targeting *Lankesterella* parasites epidemiology are needed.

It is worth highlighting that the breeding period of Acrocephalidae birds lasts from April until August at our study site; juveniles leave the nests approximately 12–14 days after hatching [36]. The juveniles sampled in this study were already independently flying, which means that they were at least one-month-old. Several PCR-positive juveniles were microscopy-positive for sporozoites. These data indicate that *Lankesterella* parasites complete their cycle and produce sporozoites in avian hosts within this period before autumnal migration. In other words, *Lankesterella* parasites are actively transmitted in Europe, and this finding corroborates with previous studies [20].

Several reports have shown the presence of *Hepatozoon* parasites in birds using exclusively morphological features of blood stages [12,13,15,16,17,18]. For instance, Merino et al. [19] used DNA sequence information and proved the presence of *Hepatozoon peircei* in storm petrels *Oceanodroma leucorhoa* and *Oceanodroma melania*, both marine bird species belonging to the Procellariiformes. This indicates that both *Lankesterella* and *Hepatozoon* parasites infect birds. However, the presence of these parasites in birds might be restricted to hosts belonging to certain taxonomic groups. In our phylogenetic analysis, the *H. peircei* sequence clustered with other *Hepatozoon* sequences as well (Figure 1). Interestingly, *H. peircei* was found infecting red blood cells of storm petrels [19], while all other previous studies reported avian *Hepatozoon* species infecting leukocytes [12]. Based on our findings, these leukocyte-inhabiting parasites might belong to the genus *Lankesterella* and not to *Hepatozoon*. However, the change of the generic position of these parasites currently would be premature, and we maintain the original binominal names of these parasites in the text. It is worth mentioning that several *Lankesterella* lineages were found infecting thrombocytes and only one infecting mononuclear leukocytes, but not red blood cells in this study. Further research is needed to clarify if the feature of host-cell identity can be used to distinguish between avian *Lankesterella* and *Hepatozoon* species in birds.

The phylogenetic analysis showed that all 16 DNA sequences obtained in the present study were closely related to other *Lankesterella* sequences from birds, which corroborates with previous studies [4,5,20]. There was a high lineage diversity in some *Lankesterella* species when targeting partial *18S* rRNA gene sequences (Figure 1, clade C). Seven different lineages were detected in *A. schoenobaenus*, the species with the highest lineage diversity in the present study. Biedrzycka et al. [5] identified two *Lankesterella* lineages in the same avian host, and Martínez et al. [20] found three lineages in *P. nivalis*. Based on the available information, it is only possible to speculate if these different lineages represent new species or just a group of lineages belonging to the same species, which is the case in several avian haemosporidian birds [37]. Only one lineage of *Lankesterella* was identified in *H. icterina*, *D. urbicum*, and *P. major*; the prevalence was low in these birds. Merino et al. [4] found only one *Lankesterella* lineage in *C. caeruleus* studied; however, the authors did not sequence all positive samples. Further studies are needed for a better understanding of the intraspecific lineage diversity in *Lankesterella* parasites.

Different lineages showed some degree of vertebrate-host specificity (clade C, Figure 1) however, their blood stages (sporozoites) were morphologically similar. Because we were dealing with wild birds and little is known about *Lankesterella* parasites in the wild, we cannot rule out the presence of co-infections of different lineages in some samples, resulting in possible amplification of only one of them using general primers. Such co-infections are common in nature, for example in haemosporidian parasites, but are not always detectable by microscopy or PCR-based methods (if the general primers are applied) [22,38,39]. This issue remains unclear in *Lankesterella* parasites. Additionally, it is necessary to mention that the nuclear *18S* rRNA is a conserved gene [40,41,42], and it might be insufficiently sensitive to distinguish between parasite species. Even though these partial gene sequences were broadly used for blood parasite barcoding, including *Hepatozoon* species, the problem of distinguishing some closely related apicomplexan species using sequences of this gene has already been reported [40,41,42]. Application of other genetic markers (for example, the mitochondrial cytochrome *b* gene) might be helpful in studies of *Lankesterella* parasite diversity. The use of other genetic markers might also help to clarify the relationship between *Lankesterella*, *Schellackia*, and *Eimeria* parasites. Few studies have reported the Lankesterellidae as a polyphyletic family, with *Lankesterella* being closer related to *Eimeria* than to *Schellackia* parasites [43,44]. In the present study we obtained similar results, however, since the number of *Eimeria* sequences included in the phylogenetic analysis was not big, it would be necessary to develop a more detailed study on this matter to draw any conclusion.

In the past, the description of new haemoparasite species was often done using the limited information about vertebrate host specificity and the morphological features of blood stages [12,13,14,15,16,17,45]. When prominent data of molecular genetics appeared, the validity of many species names was questioned, new parasites were described, and old parasites were redescribed [1]. It is important to note that old taxonomic methodologies were not always able to consider the broader biological context during species description, which, for parasites, includes peculiarities of their life cycles in vertebrate and vector species [46]. Recent molecular studies show that some avian blood parasites, which were formerly attributed to *Hepatozoon*, are genetically closer related to the *Lankesterella* parasites from amphibians [4,5,20]. The present study provided additional information to support this conclusion and added new knowledge about the persistence of sporozoites in potential vectors, without any features of the formation of syzygy, a feature of *Lankesterella*. This provided an opportunity to move the species *kabeeni* from genus *Hepatozoon* to genus *Lankesterella* and to redescribe *L. kabeeni*, as well as to develop its molecular characterization, which was lacking in the original description [13]. The phylogenetic analysis suggests that all lineages clustering in clade C (Figure 1) belong to *L. kabeeni*. Further observations are needed to prove this observation.

The only *Lankesterella* parasite DNA sequence, which was identified to a species level and available in GenBank, was attributed to *Lankesterella valsainensis* (GenBank accession DQ390207). This sequence was reported in blue tits *Cyanistes caeruleus* by Merino et al. [4], but no other information about parasite morphology is available. Because the description of *L. valsainensis* or any data about its morphology is absent, this name is invalid as a classical example of *nomen nudum* [47].

This study described two new species of *Lankesterella* parasites, i.e., *L. vacuolata* n. sp. from the common house martin *D. urbicum* and *L. macrovacuolata* n. sp. from great tits *P. major*. This is the first description of *Lankesterella* parasites in common house martin. Even though there is no *Lankesterella* species reports from *P. major*, *H. parus* was described in this avian host. If *H. parus* would be proved as belonging to *Lankesterella* in the future (see discussion above), it would be necessary to distinguish this species and *L. macrovacuolata* n. sp. The latter parasite is different from *H. parus*, particularly due to the different size of the vacuole-like structures in sporozoites (see description of *L. macrovacuolata* n. sp. for other differences).

In vitro development of *Lankesterella* parasites has never been investigated. This study shows that the great majority of sporozoites escape from infected blood cells within 1–2 h after EBA, and there are no further changes in the morphology of the extracellular forms after that. Syzygy is absent, indicating a *Lankesterella,* but not *Hepatozoon* origin. Investigation of in vitro developmental stages has been extensively used in haemosporidian research [45,46], and this study shows that it can be applied in *Lankesterella* species for a better understanding of delicate features of their biology. For example, it is known that different *Haemoproteus* species develop at different rates, and the rate of development of the same parasite species is similar, both in vivo and in vitro, at the same abiotic conditions [45,46]. This might be the case in different *Lankesterella* species and can be used for better understanding parasite biology. We call for further in vitro studies aiming to the better understand the biology of *Lankesterella* parasites.

This study shows that avian *Lankesterella* parasites persist for a long time in their potential paratenic vectors after initial infection; alive actively moving sporozoites were seen in infected mosquitoes up to 21–42 DPE in different experiments (Table 1). Additionally, experimental observations about the occurrence of *Lankesterella* spp. in *Cx. p.* forma *molestus* and *Ae. aegypti* showed that there is no development in insects. This finding corroborates with the molecular results, which suggested that these parasites belong to *Lankesterella* (the parasite persists on the sporozoite stage in vectors), but not to *Hepatozoon* (the parasite undergoes a sexual process via syzygy formation and sporogony in vectors) [4,5,20]. If *Hepatozoon* spp. would be present in our samples, the development in the invertebrate host would include morphological changes, with the formation of a syzygy and the sexual reproduction [3], instead, we could only observe the presence of sporozoites, which persist for quite a long time without any visible change in the exposed insects.

Interestingly, the mode of the *L. kabeeni* sporozoite movement, which was observed in the fresh insect midgut wet preparations in vivo, was the same as in vitro preparations used for the buffy coat diagnostics [22]. Morphologically similar sporozoites and the mode of their movement were previously reported in mosquitoes experimentally infected with amphibian *Schellackia* parasites, which also belong to Lankesterellidae [48].

It is noteworthy that the long-time persistence of *Lankesterella* sporozoites in the infected mosquitoes was reported for the first time. The ability to persist for a long time in blood-sucking insects might be an important evolutionary adaptation for parasite survival and successful transmission to a new vertebrate host. Interestingly, the mosquitoes exposed to *Lankesterella* parasites lineage LanAcro8 lived longer than the non-infected groups (Table 1). This might also increase the chances for the parasite to be successfully transmitted to new hosts. However, it should be mentioned that we collected more infected mosquitoes for dissections than controls, which resulted in a bigger number of censored data and might have contributed to the reported differences. In future mosquito survival experiments these two factors should be considered.

*Serinus canaria* were resistant to *L. kabeeni* (lineage LanDeli8), and thus cannot be recommended as experimental hosts for this blood parasite infection. This finding is in accordance with our molecular data, which indicates the presence of a certain degree of vertebrate-host specificity in some lineages (Figure 1). In other words, this study suggests that *Lankesterella* parasites from the Acrocephalidae species would not infect *S. canaria* belonging to the Fringillidae, and transmission of the same parasite species probably does not occur between birds belonging to different families. *Serinus canaria* is a relatively well investigated bird in regards of parasites [49]. Interestingly, *Lankesterella* spp. DNA sequence information is absent regarding *S. canaria* in GenBank; this is in accordance with our parasite specificity observations.

## 5. Conclusions

This study showed that the investigated intracellular avian parasites, inhabiting cells other than red blood cells, belong to *Lankesterella*, but not to *Hepatozoon*. This finding calls for a revision of the species taxonomy and systematics of avian *Hepatozoon* parasites, some of which might belong to *Lankesterella*.

Morphological features of blood stages of avian *Lankesterella* and *Hepatozoon* parasites (parasite size, parasite nucleus size and form, and morphology of vacuole-like structures in the cytoplasm) are similar and can hardly be used for distinguishing these pathogens. However, the available information suggests that avian *Lankesterella* parasites predominantly infect thrombocytes and leucocytes, while avian *Hepatozoon* spp. prefer to inhabit red blood cells. This observation needs further examination, particularly because it might be helpful for the development of these parasites’ taxonomy on species levels. Further research is needed, preferably by combining morphological and molecular analysis, as well as experimental observations in vectors and vertebrate hosts, in order to better understand biology of avian *Lankesterella* and *Hepatozoon* species.

Experimental observations show that avian *Lankesterella* parasites rapidly (within 1–2 h) escape from their blood host cell and persist for a long time (up to 42 days) in experimentally exposed mosquitoes in vivo. The long-time persistence in vectors is important epidemiologically for successful transmission.

The reported avian *Lankesterella* lineages are phylogenetically closely related; they are vertebrate-host specific on bird species levels, but hardly distinguishable, based solely on the morphology of their blood stages (sporozoites). Experimental observation showed that *L. kabeeni*, a parasite specific to Acrocephalidae species, did not infect domestic *S. canaria* belonging to the Fringillidae. In other words, this parasite was specific to birds at least on the family level. This finding is important epidemiologically.

The current knowledge about *Lankesterella* pathogens is markedly limited and remains insufficient, even on the level of prevalent data in wild birds and blood-sucking insects. Further investigation of *Lankesterella* spp. biology would allow for a better understanding of the epidemiological situation on lankesterellosis, and would clarify the role of these neglected blood parasites in regard of pathogenicity in avian hosts.

## Figures and Tables

**Table 1:** Control (non-infected) and experimentally exposed to *Lankesterella kabeeni* infection mosquitoes and their life span after blood meals.

Bird ID	Parasite-Donor Bird Species	Mosquito Species	Parasite Lineage	Parasitemia ^a^	No Exposed Insects	Maximum Life Span ^b^	Average Life Span (95% CI) ^c^
AS1	*Acrocephalus schoenobaenus*	*Aedes aegypti*	LanAcro8	12–34%	54	38	29.2 (22.7–35.7)
AS1	*A. schoenobaenus*	*Culex pipiens* forma *molestus*	LanAcro8	10–30%	28	21	19.2 (15.9–22.5)
AS2	*A. schoenobaenus*	*Aedes aegypti*	LanAcro1	8–22%	28	42	7.2 (4.9–9.6)
SC1	*Serinus canaria*	*Aedes aegypti*	Control	- ^d^	36	37	11.5 (7.1–15.9)
SC2	*S. canaria*	*Cx. p.* forma *molestus*	Control	- ^d^	32	23	8.7 (6.5–11.0)

^a^ Range of parasitemia (number of infected thrombocytes per 100 visualized thrombocytes) in different donor birds, in percentage. ^b^ Days post exposure. ^c^ 95% confidence interval. ^d^ Negative control, birds were not infected.

**Table 2:** Prevalence of *Lankesterella* parasites in adult birds of several Passeriformes families and juveniles of Acrocephalidae species.

Bird Family and Species	Overall		Adults		Juveniles	
	Examined	Positive	Examined	Positive	Examined	Positive
Acrocephalidae						
*Acrocephlus arundinaceus*	72	1 (1.4) ^a^	52	1 (1.9)	20	0 (0)
*A. dumetorum*	2	0 (0)	2	0 (0)	0	0 (0)
*A. palustris*	89	6 (6.7)	47	3 (6.4)	42	3 (7.1)
*A. schoenobaenus*	232	40 (17.2)	84	28 (33.3)	148	12 (8.1)
*A. scirpaceus*	86	5 (5.8)	35	2 (5.7)	51	3(5.9)
*Hippolais icterina*	1	1 (100)	1	1 (100)	0 (0)	0 (0)
Total	482	53 (11)	221	35 (15.8)	261	18 (6.9)
Hirundinidae						
*Delichon urbicum*	43	1 (2.3)	43	1 (2.3)	0 (0)	0 (0)
*Hirundo rustica*	101	0 (0)	101	0 (0)	0 (0)	0 (0)
*Riparia riparia*	1	0 (0)	1	0 (0)	0 (0)	0 (0)
Total	145	1 (0.7)	145	1 (0.7)	0 (0)	0 (0)
Paridae						
*Cyanistes caeruleus*	3	0 (0)	3	0 (0)	0 (0)	0 (0)
*P. major*	6	1 (16.7)	6	1 (16.7)	0 (0)	0 (0)
Total	9	1 (11.1)	9	1 (11.1)	0 (0)	0 (0)
Phylloscopidae						
*Phylloscopus collybita*	1	0 (0)	1	0 (0)	0 (0)	0 (0)
Total	1	0 (0)	1	0 (0)	0 (0)	0 (0)
Sylvidae						
*Sylvia curruca*	1	0 (0)	1	0 (0)	0 (0)	0 (0)
*S. nisoria*	1	0 (0)	1	0 (0)	0 (0)	0 (0)
*S. atricapilla*	1	0 (0)	1	0 (0)	0 (0)	0 (0)
Total	3	0 (0)	3	0 (0)	0 (0)	0 (0)
Muscicapidae						
*Erithacus rubecula*	1	0 (0)	1	0 (0)	0 (0)	0 (0)
Total	1	0 (0)	1	0 (0)	0 (0)	0 (0)
**Grand Total**	**641**	**55 (8.6)**	**380**	**37 (9.7)**	**261**	**18 (6.9)**

^a^ Percentage is given in parentheses.

**Table 3:** *Lankesterella* lineages found in adult Passeriformes birds and juveniles of the Acrocephalidae species.

Bird Family and Species	Lineages	
	Adults	Juveniles
*Acrocephalus arundinaceus*	LanAcro9 (1) ^a^	- ^b^
*A. palustris*	LanAcro5 (1) LanAcro11 (1) LanAcro12 (1)	LanAcro3 (1) LanAcro5 (2)
*A. schoenobaenus*	LanAcro1 (12) LanAcro2 (9) LanAcro6 (1) LanAcro7 (1) LanAcro8 (3) LanAcro10 (1) LanAcro13 (1)	LanAcro1 (7) LanAcro2 (5)
*A. scirpaceus*	LanAcro5 (1) LanAcro12 (1)	LanAcro4 (1) LanAcro5 (2)
*Hippolais icterina*	LanHipp1 (1)	- ^c^
*Delichon urbicum*	LanDeli1 (1)	- ^c^
*Parus major*	LanParu1 (1)	- ^c^

^a^ Number of positive individuals is given in parenthesis. ^b^ Juveniles were negative. ^c^ Juveniles were not examined.

## Data Availability

The data presented in this study are available in Supplementary material and in GenBank database (https://www.ncbi.nlm.nih.gov/genbank/, accessed on 31 March 2021) (accession numbers MW727631-MW727689).

## Supplementary Materials

The following are available online at https://www.mdpi.com/article/10.3390/ani11051451/s1, Table S1: GenBank accession numbers of partial *18S* rRNA gene sequences of vertebrate hosts and parasite species, which were used in the primers design of avian *Lankesterella*; Table S2: GenBank accession numbers of partial *18S* rRNA gene sequences and parasite species used in the phylogenetic analysis of avian *Lankesterella* (see Figure 1).
