# Supplementary material for: Lankesterella (Apicomplexa, Lankesterellidae) Blood Parasites of Passeriform Birds: Prevalence, Molecular and Morphological Characterization, with Notes on Sporozoite Persistence In Vivo and Development In Vitro
Source: Animals (Basel). 2021 May 18;11(5):1451. doi: 10.3390/ani11051451 (PMC8158525; doi:10.3390/ani11051451)
Supplement: Supplementary file 1 [file animals-11-01451-s001.zip › TableS2.pdf]

Table S2. GenBank accession numbers of partial *18S* rRNA gene sequences and parasite species used in the phylogenetic analysis of avian *Lankesterella* (see Fig. 5).

GenBank accession number	Parasite species
AF109678	*Besnoitia besnoiti*
EF210324	*Eimeria acervulina*
AF324212	*Eimeria adeneodei*
U67116	*Eimeria brunetti*
KT184338	*Eimeria dispersa*
KT184344	*Eimeria gallopavonis*
EF210322	*Eimeria máxima*
AF041437	*Eimeria meleagrimitis*
U40262	*Eimeria mitis*
KT184349	*Eimeria necatrix*
AF026388	*Eimeria tenella*
AF009245	*Frenkelia glareoli*
AF009244	*Frenkelia microti*
FJ009242	*Goussia neglecta*
FJ009241	*Goussia noelleri*
HQ224959	*Haemogregarina balli*
KM887508	*Haemogregarina pellegrini*
KF257927	*Haemogregarina stepanowi*
MK650632	*Haemoproteus columbae*
KT184369	*Hammondia hammondi*
HQ224961	*Hemolivia mariae*
KF992706	*Hemolivia mauritanica*
KP881349	*Hemolivia stellata*
AF176836	*Hepatozoon americanum*
AY461378	*Hepatozoon canis*
AF176837	*Hepatozoon catesbianae*
AY620232	*Hepatozoon felis*
JN181157	*Hepatozoon sipedon*
KF022102	*Hepatozoon peircei*
KF648870	*Isospora greineri*
AF080612	*Isospora robini*
KF648871	*Isospora superbusi*
AF080611	*Lankesterella mínima*
MF167547	*Lankesterella sp.*
MF167548	*Lankesterella sp.*
KU180248	*Lankesterella sp.*
MF167544	*Lankesterella sp.*
MF167555	*Lankesterella sp.*
MF167552	*Lankesterella sp.*
MF167554	*Lankesterella sp.*
KJ131417	*Lankesterella sp.*
MF167545	*Lankesterella sp.*
MF167546	*Lankesterella sp.*
MF167549	*Lankesterella sp.*
MF167551	*Lankesterella sp.*
MF167550	*Lankesterella sp.*
MF167553	*Lankesterella sp.*
MG808272	*Lankesterella sp.*
MG808274	*Lankesterella sp.*

MG808273	*Lankesterella sp.*
DQ390207	*Lankesterella valsainensis*
MK650741	*Leucocytozoon sp.*
MK650564	*Plasmodium elongatum*
MK650600	*Plasmodium homocircumflexum*
MK650484	*Plasmodium relictum*
AF017121	*Sarcocystis buffalonis*
AF017120	*Sarcocystis cruzi*
AF017122	*Sarcocystis hirsuta*
AF006470	*Sarcocystis hominis*
KJ131415	*Schellackia bolivari*
KJ131414	*Schellackia orientalis*
L37415	*Toxoplasma gondii*
