# Supplementary material for: Lankesterella (Apicomplexa, Lankesterellidae) Blood Parasites of Passeriform Birds: Prevalence, Molecular and Morphological Characterization, with Notes on Sporozoite Persistence In Vivo and Development In Vitro
Source: Animals (Basel). 2021 May 18;11(5):1451. doi: 10.3390/ani11051451 (PMC8158525; doi:10.3390/ani11051451)
Supplement: Supplementary file 1 [file animals-11-01451-s001.zip › TableS1.pdf]

Table S1. GenBank accession numbers of partial *18S* rRNA gene sequences of vertebrate hosts and parasite species, which were used in the primers design of avian *Lankesterella*.

GenBank accession number	Organism (vertebrate host or parasite species)
FJ688019	*Acipenser fulvescens*
AF494058	*Adelina bambarooniae*
DQ096835	*Adelina dimidiata*
DQ096836	*Adelina grylli*
AF173605	*Alligator mississippiensis*
AF173614	*Anas platyrhynchos*
MH304758	Apicomplexa
MH304760	Apicomplexa
LK077046	*Apteryx australis*
AF173619	*Apus affinus*
DQ227420	*Besnoitia besnoiti*
JF314861	*Besnoitia besnoiti*
AF173620	*Bubo virginianus*
MG590304	*Caloenas nicobarica*
KT184332	*Caryospora* cf.
KT184331	*Caryospora* cf.
AF173638	*Charadrius semipalmatus*
AF173622	*Chordeiles acutipennis*
HM117907	Coccidia
HM117908	Coccidia
AF173630	*Columba livia*
AF173625	*Coracias caudata*
AF173611	*Coturnix pectoralis*
AJ311672	*Crocodylus niloticus*
AY954885	*Cryptosporidium andersoni*
DQ060422	*Cryptosporidium andersoni*
DQ060421	*Cryptosporidium baileyi*
EU162754	*Cryptosporidium fragile*
EU162752	*Cryptosporidium fragile*
AF093497	*Cryptosporidium muris*
AB089284	*Cryptosporidium muris*
X64343	*Cryptosporidium muris*
AY642591	*Cryptosporidium muris*
X64341	*Cryptosporidium parvum*
X64340	*Cryptosporidium parvum*
AF093499	*Cryptosporidium serpentis*
AF093501	*Cryptosporidium serpentis*
AF093500	*Cryptosporidium serpentis*
AF513227	*Cryptosporidium* sp.
AF173628	*Cuculus pallidus*
KT184368	*Cystoisospora canis*
AY618555	*Cystoisospora* cf
AB519675	*Cystoisospora* sp.
AY279205	*Cystoisospora timoni*
MG590305	*Didunculus strigirostris*

DQ538351	*Eimeria acervulina*
EF210323	*Eimeria acervulina*
KC305176	*Eimeria adenoeides*
KC305170	*Eimeria adenoeides*
KC305180	*Eimeria adenoeides*
AB769547	*Eimeria alabamensis*
AY613853	*Eimeria arnyi*
AB769566	*Eimeria auburnensis*
AB769563	*Eimeria auburnensis*
AB769583	*Eimeria bovis*
AB769600	*Eimeria bukidnonensis*
AB769602	*Eimeria canadensis*
MF860827	*Eimeria* cf.
MF860826	*Eimeria* cf.
FJ236374	*Eimeria* cf.
KT184339	*Eimeria falciformis*
KT184341	*Eimeria gallopavonis*
KC305187	*Eimeria meleagrimitis*
KT184350	*Eimeria papillata*
HM117016	*Eimeria* sp.
AB769636	*Eimeria subspherica*
AF026388	*Eimeria tenella*
DQ136183	*Eimeria tenella*
GU479674	*Eimeria variabilis*
AB769647	*Eimeria wyomingensis*
AB769657	*Eimeria zuernii*
AB769659	*Eimeria zuernii*
KT956976	*Eimeriidae*
NR46271	*Equus caballus*
AF009245	*Frenkelia glareoli*
AF173624	*Galbula pastazae*
KT445934	*Gallus gallus*
MG590306	*Goura cristata*
MG590323	*Goura victoria*
AY043206	*Goussia janae*
MH758784	*Goussia* sp.
MH758783	*Goussia* sp.
GU479656	*Goussia szekelyi*
AF173632	*Grus canadenses*
HQ224959	*Haemogregarina balli*
KF854253	*Hammondia hammondi*
KP881349	*Hemolivia stellata*
MG758136	*Hepatozoon banethi*
MG758137	*Hepatozoon banethi*
MG758133	*Hepatozoon banethi*
AY461375	*Hepatozoon canis*
AY461376	*Hepatozoon canis*
KX712128	*Hepatozoon canis*
LC169075	*Hepatozoon canis*

KU893123	*Hepatozoon canis*
KU893125	*Hepatozoon canis*
KU893121	*Hepatozoon canis*
EU289222	*Hepatozoon canis*
AY731062	*Hepatozoon canis*
HQ224954	*Hepatozoon* cf.
HQ224962	*Hepatozoon* cf.
HQ224963	*Hepatozoon* cf.
MG593275	*Hepatozoon ewingi*
KX017290	*Hepatozoon felis*
MG041594	*Hepatozoon involucrum*
MG041591	*Hepatozoon involucrum*
MG041593	*Hepatozoon involucrum*
MG041592	*Hepatozoon involucrum*
MG136687	*Hepatozoon martis*
JN181157	*Hepatozoon sipedon*
KF022102	*Hepatozoon peircei*
MF541372	*Hepatozoon* sp.
AB181504	*Hepatozoon* sp.
FJ719818	*Hepatozoon* sp.
FJ719817	*Hepatozoon* sp.
FJ719815	*Hepatozoon* sp.
FJ719816	*Hepatozoon* sp.
FJ719819	*Hepatozoon* sp.
AY600626	*Hepatozoon* sp.
AY600625	*Hepatozoon* sp.
JX644997	*Hepatozoon* sp.
JX644996	*Hepatozoon* sp.
KU680465	*Hepatozoon* sp.
KU680466	*Hepatozoon* sp.
KU680461	*Hepatozoon* sp.
KJ413113	*Hepatozoon* sp.
KJ413132	*Hepatozoon* sp.
JX644998	*Hepatozoon* sp.
FJ719813	*Hepatozoon* sp.
FJ719814	*Hepatozoon* sp.
MH174343	*Hepatozoon* sp.
EF222259	*Hepatozoon* sp.
EF222257	*Hepatozoon* sp.
KU198330	*Hepatozoon* sp.
LC169077	*Hepatozoon* sp.
HQ224960	*Hepatozoon* sp.
MG519502	*Hepatozoon* sp.
MG519501	*Hepatozoon* sp.
MG519504	*Hepatozoon* sp.
KU680463	*Hepatozoon* sp.
KU680462	*Hepatozoon* sp.
AY461377	*Hepatozoon* sp.
MG041596	*Hepatozoon tenuis*

MG041597	*Hepatozoon tenuis*
MG041595	*Hepatozoon tenuis*
MG041599	*Hepatozoon tenuis*
MG041602	*Hepatozoon thori*
MG041600	*Hepatozoon thori*
MG041601	*Hepatozoon thori*
MF164258	*Homo sapiens*
DQ060658	*Isospora belli*
AF106935	*Isospora belli*
L76471	*Isospora felis*
AF080612	*Isospora robini*
HQ224955	*Klossia helicina*
HQ224956	*Klossia helicina*
MG808274	*Lankesterella* sp.
MG808272	*Lankesterella* sp.
MG808273	*Lankesterella* sp.
DQ390207	*Lankesterella valsainensis*
AF173637	*Larus glaucoides*
AY217921	*Melanoseps occidentalis*
AJ419877	*Meleagris gallopavo*
AF173618	*Musophaga porphyreolopha*
AJ271354	*Neospora caninum*
U16159	*Neospora caninum*
EU334134	*Nephroisospora eptesici*
AF173606	*Nothoprocta ornata*
AF173613	*Ortalis guttata*
AY305326	*Perkinsus andrewsi*
AF102171	*Perkinsus andrewsi*
AF509333	*Perkinsus atlanticus*
KX514016	*Perkinsus olseni*
KX513987	*Perkinsus olseni*
AF252288	*Perkinsus* sp.
L07375	*Perkinsus* sp.
AF173615	*Picoides pubescens*
EU282018	*Sarcocystis alces*
MF596236	*Sarcocystis arctica*
MF596228	*Sarcocystis arctica*
MF596220	*Sarcocystis arctica*
KX022102	*Sarcocystis arctica*
MF039330	*Sarcocystis arieticanis*
MH413035	*Sarcocystis arieticanis*
KT901132	*Sarcocystis bovifelis*
KT901153	*Sarcocystis bovini*
KT901142	*Sarcocystis bovini*
KY019021	*Sarcocystis capreolicanis*
KY019027	*Sarcocystis capreolicanis*
JN256129	*Sarcocystis capreolicanis*
KY019024	*Sarcocystis capreolicanis*
KY019023	*Sarcocystis capreolicanis*

JN226118	*Sarcocystis capreolicanis*
KY973342	*Sarcocystis cervicanis*
KY973339	*Sarcocystis cervicanis*
KY973333	*Sarcocystis cervicanis*
HM125054	*Sarcocystis columbae*
LC171829	*Sarcocystis cruzi*
JX679467	*Sarcocystis cruzi*
GQ251013	*Sarcocystis elongata*
MF596181	*Sarcocystis entzerothi*
MF596180	*Sarcocystis entzerothi*
KX643337	*Sarcocystis entzerothi*
KY019030	*Sarcocystis gracilis*
JN256131	*Sarcocystis gracilis*
FJ196261	*Sarcocystis gracilis*
JN256128	*Sarcocystis hjorti*
AY015113	*Sarcocystis lacertae*
JQ733508	*Sarcocystis lari*
KU247921	*Sarcocystis levinei*
KU247914	*Sarcocystis levinei*
KY973357	*Sarcocystis linearis*
KY973361	*Sarcocystis linearis*
KY973365	*Sarcocystis linearis*
KY019041	*Sarcocystis linearis*
KY019035	*Sarcocystis linearis*
KY019045	*Sarcocystis linearis*
KY973358	*Sarcocystis linearis*
KY019053	*Sarcocystis linearis*
MF596216	*Sarcocystis lutrae*
KY973375	*Sarcocystis morae*
KY973379	*Sarcocystis morae*
M64244	*Sarcocystis muris*
EF467655	*Sarcocystis rangi*
GQ250978	*Sarcocystis rangiferi*
GQ250985	*Sarcocystis rangiferi*
HM185742	*Sarcocystis rileyi*
EU282021	*Sarcocystis scandinavica*
EU282025	*Sarcocystis scandinavica*
EU282026	*Sarcocystis scandinavica*
KY019060	*Sarcocystis silva*
KY019058	*Sarcocystis silva*
JN256132	*Sarcocystis silva*
KY019063	*Sarcocystis silva*
KT901096	*Sarcocystis sinensis*
KT901106	*Sarcocystis sinensis*
KT901105	*Sarcocystis sinensis*
KT901103	*Sarcocystis sinensis*
KT901108	*Sarcocystis sinensis*
GQ245670	*Sarcocystis* sp.
AB691780	*Sarcocystis* sp.

KF309698	*Sarcocystis* sp.
AB257162	*Sarcocystis* sp.
JN256133	*Sarcocystis* sp.
KF831281	*Sarcocystis taeniata*
KF831292	*Sarcocystis taeniata*
KF831282	*Sarcocystis taeniata*
EF056012	*Sarcocystis tarandivulpes*
EF467657	*Sarcocystis tarandivulpes*
KP263758	*Sarcocystis tenella*
KP263753	*Sarcocystis tenella*
GQ251022	*Sarcocystis truncata*
GQ251029	*Sarcocystis truncata*
KY973325	*Sarcocystis venatoria*
KY973324	*Sarcocystis venatoria*
KY973328	*Sarcocystis venatoria*
KU244524	*Sarcocystis zamani*
AJ419876	*Struthio camelus*
NR46261	*Sus scrofa*
AF173626	*Tockus nasutus*
EF472967	*Toxoplasma gondii*
KX008020	*Toxoplasma gondii*
KX008008	*Toxoplasma gondii*
AF173623	*Trogon collaris*
AF173631	*Turnix sylvatica*
AF173616	*Tyrannus tyrannus*
KP404891	Uncultured eukaryote
KP404707	Uncultured eukaryote
KP404652	Uncultured eukaryote
AF173627	*Upupa epops*
AF173617	*Urocolius macrourus*
X02995	*Xenopus laevis*
